# Does breast oncoplastic surgery improve quality of life?

**DOI:** 10.3389/fonc.2022.1099125

**Published:** 2023-01-12

**Authors:** René Aloisio da Costa Vieira, Antônio Bailão-Junior, Idam de Oliveira-Junior

**Affiliations:** ^1^ Programa de Pós-Graduação em Tocoginecologia, Faculdade de Medicina de Botucatu, Botucatu/SP, Brazil; ^2^ Programa de Pós-Graduação em Oncologia, Hospital de Câncer de Barretos, Barretos/SP, Brazil; ^3^ Departamento de Cirurgia Oncológica, Divisão de Mastologia, Hospital de Câncer de Muriaé, Muriaé/MG, Brazil; ^4^ Active Member of European Organisation for Research and Treatment (EORTC) Quality of life Group, Brussels, Belgium; ^5^ Departamento de Mastologia e Reconstrução Mamária, Hospital de Câncer de Barretos, Barretos/SP, Brazil

**Keywords:** quality of life, systematic review, meta-analysis (MA), breast cancer, oncoplastic surgery, patient-reported outcome measures

## Abstract

Breast Oncoplastic Surgery (OS) has established itself as a safe procedure associated with the treatment of breast cancer, but the term is broad, encompassing procedures associated with breast-conserving surgeries (BCS), conservative mastectomies and fat grafting. Surgeons believe that OS is associated with an increase in quality of life (QOL), but the diversity of QOL questionnaires and therapeutic modalities makes it difficult to assess from the patient’s perspective. To answer this question, we performed a search for systematic reviews on QOL associated with different COM procedures, and in their absence, we selected case-control studies, discussing the main results. We observed that: (1) Patients undergoing BCS or breast reconstruction have improved QoL compared to those undergoing mastectomy; (2) In patients undergoing BCS, OS has not yet shown an improvement in QOL, a fact possibly influenced by patient selection bias; (3) In patients undergoing mastectomy with reconstruction, the QoL results are superior when the reconstruction is performed with autologous flaps and when the areola is preserved; (4) Prepectoral implants improves QOL in relation to subpectoral implant-based breast reconstruction; (5) ADM do not improves QOL; (6) In patients undergoing prophylactic mastectomy, satisfaction is high with the indication, but the patient must be informed about the potential complications associated with the procedure; (7) Satisfaction is high after performing fat grafting. It is observed that, in general, OS increases QOL, and when evaluating the procedures, any preservation or repair, or the use of autologous tissues, increases QOL, justifying OS.

## Introduction

The World Health Organization (WHO) (1) defines quality of life (QOL) as “the individual’s perception of their position in life, in the context of culture, value systems in which they live in relation to their goals, expectations, standards and concerns”. In patients treated for breast cancer, many of the acute symptoms disappear. However, emotional deficits in social relationships and cognitive functions, associated with specific symptoms and concerns arising from cancer, impair QOL (2).

In the past, the only surgical treatment for breast cancer was mastectomy, with the possibility of late reconstruction with a myocutaneous flap (3). Subsequently, breast-conserving surgery (BCS) was established when combined with radiotherapy (4, 5). However, it is not always associated with good cosmetic results, as up to 30% of patients undergoing quadrantectomy require delayed repair due to unsatisfactory aesthetic results (6).

Thus, the concept of oncoplastic surgery (OS) is born, which is defined as the use of plastic surgery techniques to improve the aesthetic result of oncological surgery (7, 8). The surgery can be performed after mastectomy or BCS (9), with increasing indications in clinical practice. From an oncological point of view, greater ease of wide resection is observed, with the possibility of wider margins (10), a lower index of compromised margins (11) and a greater amount of resected tissue, without aesthetic damage (12).

Many patients who are not initially candidates for BCS (13) can undergo this procedure with the help of OS (14, 15), especially in the presence of tumours larger than 5 centimetres and with localized skin infiltration and multifocal and multicentric tumours, provided that it is possible to obtain neoplasia-free margins and that the residual breast volume allows an aesthetically satisfactory result. In this sense, the concept of extreme oncoplasty (EO) emerges (16).

Likewise, radical mastectomies have become more conservative, through skin preservation, with skin-sparing mastectomy (SSM) and nipple-sparing mastectomy (NSM) (17, 18). Immediate breast reconstruction, initially performed with myocutaneous flaps, was mostly replaced by the use of prostheses, and was considered a safe procedure, given the low cancer recurrence rates (19–22) and the high degree of patient satisfaction (23).

However, OS is generally used to describe a broad group of surgeries associated with BCS, including mastectomy with immediate reconstruction and late reconstruction surgeries (7, 8). Thus, when evaluating OS, we must consider the type of surgery, the conditions associated with its indication, the cosmetic quality and the QOL of the patient (24, 25). OS seems to be associated with the improvement of QOL (8). The articles usually assess specific situations and little studies evaluate all situations associated with OS and QOL (26–28). In this study, we sought to identify the main circumstances leading to OS, QOL questionnaires, systematic reviews and case−control studies.

### Quality-of-life questionnaires

Several questionnaires can be found in the literature, but they need to be validated through a specific methodology (29, 30). The patient-reported outcome measures (PROMs) are organized in domains and questions. Domains correspond to the grouping of questions that evaluate the same subject. Grouping similar situations allows us to consider the subject and compare groups of patients in similar situations.

QOL associated with breast reconstruction were validated (26, 31, 32). To better understand and value the QOL questionnaires, we must understand how they are created (33, 34), the importance of the domains, the validation studies (35) and the steps associated with linguistic translation (36, 37).

The construction of a QOL questionnaire involves four main phases (34). The first phase is the question generation phase. Patients at different stages of the disease and health professionals are interviewed to determine the main questions to be asked. In the second phase, a list of questions is created, measurement scales are evaluated, health professionals are consulted, and an initial version of the questionnaire is drafted. A smaller group of patients evaluates questions for redundancy and low response rates, decreasing the number of questions, organizing the potential domains and drafts a potential questionnaire. In the third phase, the questionnaire is administered to a group of patients, and a validation test is performed. The acceptability, the structure of the questionnaire and the variability are evaluated, and changes are suggested. In the fourth phase, the module is completed, and validation tests are performed, aiming at a final review, regarding the number of questions and domains, and reaching a final version (33, 34).

There are several steps associated with the validation of a QOL questionnaire (38, 39). To validate a QOL questionnaire, in general, construct validity and reliability can be evaluated. Validity is the ability of a test to measure what it is proposed to measure. Factor analysis group questions organizing the domains. The internal consistency is assessed, which evaluates the degree of uniformity or coherence between the responses of the subjects to each of the domains that make up the instrument. In addition, the test-retest is performed, which evaluates the reliability at two different times, when no changes in the disease are observed. Construct validity evaluates the construction of the questionnaire through known, convergent or divergent (discriminant) groups and factor analysis. In the convergent validation of the scale, the correlations between the questionnaires and the conceptually related measures are evaluated, and they are expected to be substantially related to each other. For this purpose, domains of different QOL questionnaires are compared. This method allows the separation of the domains of the original questionnaire and tests whether the relationship of the original scale will be confirmed between other QOL and the variables observed in another language. Reliability evaluates whether the instrument is reliable and measures a construct over time between different individuals and situations. Confirmatory factor analysis is used to test the hypothetical scale structure and cross-cultural equivalence of the measurement properties.

A questionnaire is valid in the language in which it was created. To be used in another language, it must be translated, and the questions must have the same meaning in the translated language. There are different methods of translation and cultural adaptation (36, 37). Briefly, considering an example of translation into Portuguese/Brazil (36, 40), we have the following: translation from English to Portuguese/Brazil, by native Brazilians with English skills; synthesis of the translation by an expert committee; reverse translation into English; evaluating the versions by a committee of experts, comparing the versions and arriving at the initial version for the Portuguese language; pretest with 10 patients, aiming to evaluate understanding, eliminate embarrassing items, respond to semantic questions, adapt the questions, and test them with a small number of patients, reaching the final Portuguese/Brazil version (40).

### Quality-of-life questionnaires associated with breast cancer

There are several QOL questionnaires related to cancer. In the literature, we found questionnaires used to evaluate cancer in general, questionnaires associated with specific situations (such as anxiety and depression), specific questionnaires for breast cancer and questionnaires developed to evaluate surgical results. In addition, there were questionnaires used for other pathologies, which can be used in breast cancer given the sequelae associated with treatment. In the context of cancer, there are reviews on the subject (29, 32), and the most used in breast cancer studies are those presented in **Table 1**. Of these, the EORTC QLQ-BR45 (41) (update of EORTC QLQ-BR23) is in Phase 4 of validation.

**Table 1:** Main questionnaires associated with breast cancer treatment.

Quality of Life	Type of evaluation
General	EORTC QLQ-30; FACT-G
Breast	FACT-B; EORTC QLQ-BR23; EORTC QLQ-BR45 (BR23 actualization)
Brest Surgery	Mastectomy: BREAST-Q/mastectomy module
	Breast-conserving Surgery: BCTOS; BREAST-Q/breast conserving surgery module
	Reconstruction: MBROS, EORTC BRECON23; BREAST-Q reconstruction module
Shoulder	FACT-B+4; SPADI; DASH; Quick-DASH
Special situation	Anxiety and depression (HADS)

EORTC, European Organisation for Research and Treatment; QLQ, quality of life questionnaire; FACT, Functional Assessment of cancer therapy; BCTOS, Breast Cancer Treatment Outcome Scale; BREAST-Q, Breast questionnaire; MBROS, Michigan Breast Reconstruction Outcomes Study; BRECON23, breast reconstructive; SPADI, Shoulder Pain and Disability Index; DASH, Disabilities of the Arm, Shoulder and Hand; HADS, Hospital Anxiety and Depression Scale

### Quality-of-life questionnaires associated with breast surgery

Despite the existence of general questionnaires for breast cancer, questionnaires were created to evaluate the relationship between the type of surgery and QOL (32).

For patients undergoing BCS, we have the BCTOS (*Breast Cancer Treatment Outcome Scale*) *(* 35, 40) and the Breast-Q module for BCS (42). The BCTOS, when formulated, used another methodology for the construction of questionnaires, and for a long time it was the only questionnaire associated with BCS. The 22-item survey subjectively evaluates the aesthetic and functional outcomes after breast cancer treatment through questions about functional status, cosmetic status, specific breast pain and oedema (43). In patients undergoing BCS combined with radiotherapy, BCTOS was effective (44). It was observed, through the BCTOS, that the specific breast pain related to the treatment exceeds the importance of the cosmetic result in relation to QOL. Nevertheless, the appearance of the breast after conservation surgery is significantly associated with psychosocial outcomes, and women with large breast asymmetry are more likely to have a worse psychosocial state than those with minimal asymmetry (43). Regarding the Breast-Q, the initial questionnaire for plastic surgery was used, and recently a version of the BCS was created (42).

For patients who have undergone mastectomy and breast reconstruction, historically, the MBROS (32), followed by the Breast-Q reconstruction module (45) and EORTC QLQ BRECON23 (46) have been used. Using MBROS, delayed reconstruction increases emotional well-being and body image. Immediate and delayed breast reconstruction provide substantial psychosocial benefits for mastectomy patients, but the type of reconstruction did not impact in QOL (47). BRECON23 was published in 2018 and uses new methodology for QOL development. It is divided in scales are related to surgical side-effects, sexuality, satisfaction (breast cosmesis, nipple cosmesis, surgery), donor-site symptoms and single items (46). The number of publications using MBROS and BRECON are low.

New methodologies and validation studies were used for Breast-Q and BRECON questionnaires (31). The Breast-Q, initially developed for the evaluation of plastic and reconstructive breast surgery (48), is divided into six domains: satisfaction with the breasts, overall outcome, care processes, psychosocial, physical and sexual. In the first version, questionnaires related to augmentation mammoplasty, reduction mammoplasty and reconstruction were created (48). Currently, the Breast-Q is in its the second edition and is the most popular questionnaire for breast reconstruction (49, 50). Regarding the Breast-Q, the main domains related to cancer modules are summarized in **Table 2**.

**Table 2:** Summary of domains and number of questions in BREAST-Q version 2.0 for breast cancer.

	BCS		Mastectomy		Reconstruction	
	Pre	Post	Pre	Post	Pre	Post
**DOMAIN OF QUALITY OF LIFE**						
**Psychosocial well-being**	10	10	10	10	10	10
**Sexual well-being**	6	6	6	6	6	6
Physical well being						
- Chest	10	9	10	11	10	11
- Abdomen	–	–	–	–	4	7
**Adverse effects of radiation**	–	6	–	6	–	6
SATISFACTION DOMAINS						
**Satisfaction with Breast**	4	11	4	4	4	15
Satisfaction with the Results						
- Nipple reconstruction	–	–	–	–	–	1
- Abdomen	–	–	–	–	1	3
- Implant	–	–	–	–	–	2
Satisfaction with care						
Satisfaction with Information						
- Surgeon	–	12	–	12	–	15
- Radiotherapist	–	11	–	–	–	–
Satisfaction experience						
- Surgeon	–	12	–	12	–	12
- Medical Team	–	7	–	7	–	7
- Office Staff	–	7	–	7	–	7
Satisfaction with Latissimus Dorsi						
Back	–	–	–	–	8	8
Back and shoulder	–	–	–	–	11	11
**Sub-total**	30	91	30	75	35	102
RECONSTRUCTION EXPECTATIONS						
Short form	–	–	–	–	5 questions	–
Long form	–	–	–	–	27 questions	–

BCS, Breast- conserving Surgery.

## Quality of life and oncoplastic surgery

To evaluate QOL in OS setting, we performed a literature review in PubMed database. We choose the terms: Breast Neoplasms [Mesh] and (“Surgery, Plastic”[Mesh] or “oncoplastic surgery” or “oncoplasty” or “oncoplastic” or “Reconstructive Surgical Procedures”[Mesh] or “Mammaplasty”[Mesh] or “Mastectomy, Segmental”[Mesh]) and “Quality of Life”[Mesh]. The terms were evaluated (09/12/2022) without (n=926) or with filters (Meta-analysis, Review and Systematic Review; n=127). Based on title and resume, 25 articles were selected. In the absence of review articles, case-control studies or observational studies were evaluated.

OS has become a generic term. With regard to BCS, the concept goes beyond reduction mastoplasty techniques, with different techniques aimed at the readjustment of breast tissue to contralateral symmetrisation. Char et al (26) performed a systematic review of QL in level 2 volume displacement or volume replacement OS, including NSM, SSM with autologous or IBBR. The studies used Breast-Q or other validated PROMs. Of the 702 initial articles, 43 were included, representing 14,994 patients, and the main questionnaire used was the Breast-Q (n = 11,176). Using Breast-Q, 1,400 patients who underwent BCS and OS, 2,970 who had reconstruction with autologous flap and 6,806 who received implants were selected. Superior results were observed in the BCS in relation to mastectomy with implant, in autologous reconstruction in relation to the implant, in nipple preservation in relation to the absence of nipple preservation, and in the use of the prepectoral implant in relation to the retro-pectoral (26).

Many studies have compared specific surgical situations of OS and its relationship with QOL. We then sought, through systematic reviews, to choose more representative studies that evaluated OS in different surgical situations related to the treatment of breast cancer. In the absence of systematic reviews case-control studies or case series were selected for discussion.

### Conservative breast treatment and oncoplastic surgery

The relationship between BCS and OS involves several criteria, ranging from indication (tumour, patient, safety), type of surgery (technique, oncological safety, laterality, symmetrisation, follow-up time), and cosmetic quality, influencing QOL (24). QOL, in turn, is influenced by conditions associated with treatment, reflected in the sequelae, return to usual activities, whether in relation to work, family or sexuality.

A systematic review that evaluated the topic compared BCS associated or not with OS. Of the 688 initial articles, 6 were selected, which included 832 patients with controversial results; OS was not associated with QOL improvement in 5 studies, and an association with improvement was observed in only one study (51). The nature of the studies, usually retrospective, the patient selection bias, the time since the performance of the primary procedure and the absence of systematic use of symmetrisation in all patients negatively influenced the results of patients undergoing OS-BCS (25, 52).

With regard to BCS associated with EO (16), there is only one study in the literature (53), which analysed 204 patients, only 33 of whom had undergone EO. The results were superior when performing EO in the face of psychological well-being and satisfaction with the breast, outcome and nipple-areola complex (53).

### Mastectomy and oncoplastic mastectomy

The history of breast reconstruction begins with late reconstructions using myocutaneous flaps (autologous) and changed over time to immediate reconstructions in which the flaps were replaced by breast prostheses. We proceeded to skin-preserving mastectomies, followed by nipple-preserving mastectomies and then prophylactic mastectomies. All of these surgeries have pros and cons. Over time, asymmetries and adverse effects became more pronounced, especially in the presence of radiotherapy. Recently, to refine the results, we resorted to fat grafting.

Breast loss, without shape replacement, implies a decrease in QOL. Meta-analysis evaluated the QOL of patients undergoing mastectomy without reconstruction compared to patients undergoing BCS (54). Initially, 892 articles were evaluated, and 6 including a total of 1,931 patients were selected. It was found that patients undergoing BCS have better body image and future prospects, with a decrease in the effects associated with local effects.

BCS or reconstruction is always better than mastectomy without reconstruction. The role of the presence of breast reconstruction in relation to mastectomy was evaluated. A review of 277 studies, 9 were identified and 1.734 analysed, observing that the absence of reconstruction was associate with increased risk of depression (55). A study conducted with 400 patients using Breast-Q evaluated four groups (control, BCS, mastectomy with and without reconstruction), observing better satisfaction with breast appearance and sexual wellbeing in patients undergoing reconstructive mastectomy, followed by BCS and mastectomy without reconstruction. When evaluating the BCS in relation to the control group, the results were similar in relation to breast satisfaction, but the sexual wellbeing was superior in the control group (56). Another study evaluated 618 patients divided into BCS, mastectomy, and mastectomy with reconstruction groups using the EORTC QLQ-C30 and QLQ-BR23 questionnaires. Similar results were observed for role functioning and social functioning in patients undergoing BCS and mastectomy with reconstruction. However, when evaluating body image, the results were higher in patients undergoing BCS, followed by patients undergoing mastectomy with reconstruction compared to patients undergoing mastectomy without reconstruction (57). A meta-analysis comparing BCS versus mastectomy evaluated 9 studies identify 2.301 patient, observing better QV associated to BCS in relation to body image, emotional function and social function (58).

A systematic review and meta-analysis evaluated the impact of breast reconstruction in relation to BCS. From 12,192 initial articles, there were 16 articles, with the analysis of 5,544 patients (1,458 mastectomies, 2,612 undergoing BCS and 1,474 undergoing mastectomy with reconstruction). The results showed great heterogeneity among the studies, with similar results in relation to BCS and mastectomy with reconstruction. In turn, the patients who underwent mastectomy without reconstruction exhibited poorer physical health and body image (27).

The role of the presence of breast reconstruction in relation to mastectomy was evaluated. From 277 studies, 9 were identified and 1.734 analysed, observing that the absence of reconstruction was associate with increased risk of depression (55).

Platt et al (59), performed a review of different conditions related to mastectomy with breast reconstruction and QV, reporting: (1) Immediate and delayed breast reconstruction increases satisfaction and quality of life; (2) Autologous reconstruction demonstrates superior PROMs over long-term when compared with implant-based breast reconstruction (IBBR); (3) NSM was associated with increased satisfaction than SSM.

Saldanha et al (28) performed a systematic review of breast reconstruction after mastectomy, selecting 83 nonrandomized studies, 8 randomized controlled trials and 69 single group studies. They observed that: (1) autologous reconstruction were associated with clinically better patient satisfaction with breast and sexual well-being than IBBR; (2) There is insufficient evidence about IBBR versus radiotherapy and QV; (3) Silicone and saline implants result in clinically similar patient satisfaction; (3) The evidence related to acellular dermal matrix (ADM) and QV is insufficient; (4) The type of Autologous reconstruction did not influence QV.

Also, in the comparison between the different breast reconstruction modalities (autologous tissue or implant), a meta-analysis performed on 219 initial studies yielded 9 studies suitable for analysis, encompassing 2,954 patients (2,129 with implants and 825 with autologous tissue). High overall satisfaction was observed among patients who had undergone breast reconstruction, and overall satisfaction and satisfaction with the breast was higher with the use of autologous tissue. On the other hand, psychosocial, psychic and sexual well-being was higher in patients with breast implants (60). Another meta-analysis evaluated the same item, selecting from 280 articles, 10 full-texts, including a total of 4,957 patients, of which 3,836 were evaluated using the Breast-Q questionnaire. It was found that satisfaction with the results, the breasts and sexual well-being was higher with the use of autologous tissue (61).

The use of prepectoral or subpectoral IBBR was evaluated. A meta-analysis evaluates 3.789 studies, 7 publications and 548 patients, observing that patients with prepectoral implants reported higher Breast-Q scores and lower postoperative pain (62), suggesting the use of acellular dermal matrix (ADM). Although, the use of acellular dermal matrix (ADM) do not improve the QOL (63), and one or two-stage IBBR with ADM, also do not chance QOL (64).

Nipple-areolar preservation improves QOL, although the number of studies and casuistry are limited, positive results were observed (59). Char et al (26) performed a systematic review of QL including NSM and SSM with autologous or IBBR, observing superior results related do NSM. A non-randomized, cross-sectional study used evaluated the impact of areolar preservation with Breast-Q questionnaire. It evaluated 137 patients (83 SSM x 53 NSM), observing that body image and sexual functioning associated to SSM (65). Wei et al, prospective evaluate patients submitted to NSM (n=52) and patients submitted do SSM and areolar reconstruction (202). NSM patients reported higher scores in psychosocial and sexual well-being (66). Two studies performed matched comparison, all using Breast-Q. The first (n = 62), matched by reconstruction type and operative period, compared NSM (n = 32) and SSM (n = 32), observing better satisfaction of results and the breasts in NSM (67). The second, with smaller number of patients (n = 52; 26x26), matched by age, race and body index, observed a significant improvement only in sexual well-being, associated with NSM (68).

Prophylactic mastectomy has risen in popularity, a fact associated with the dissemination of genetic tests, facilitating the selection of patients. It is observed that the patients are satisfied with the indication, but have complaints related to the prosthesis (69, 70). The first systematic review found 1,082 studies and selected 22 studies with a total of 2,046 patients. Satisfaction with the indication, high psychosocial well-being and body image were observed, with social well-being and somatosensory function being the most affected items (69). The second review, based on 7,272 articles, selected 7 articles that included 730 women and used different questionnaires. Overall satisfaction and cosmetic results were high, but surgery was associated with complaints related to breast hardness, numbness and sex, suggesting the importance of informing patients about the complications associated with the procedure (70).

Contralateral prophylactic mastectomy for unilateral breast cancer was evaluated in a systematic review of 19 articles, representing 6.088 patients. High levels of satisfaction were observed with the decision for surgery, with high satisfaction with cosmesis and reconstruction (71).

Evaluating robotic mastectomy, one study (n=80), using Breast Q, evaluated the impact in QOL, observing that Breast-Q scores in satisfaction with breasts, psychosocial, physical and sexual well-being were significantly higher after robotic mastectomy in relation to conventional mastectomy (72).

To improve the cosmetic results, we have fat grafting. A systematic review evaluating the technique found 2,915 articles and selected 6 that reported on Breast-Q, representing 1,437 patients. Although fat grafting improves breast satisfaction, the difference was not significant (73).

## Discussion

When evaluating studies that selected patients for OS, it is necessary to consider that most of them are retrospective, and even in prospective patients, there may be a selection bias. Commonly, patients subjected to OS have a large tumour size, are younger, undergo neoadjuvant chemotherapy, have higher education, or are potentially more demanding, not accepting major defects or mastectomy without reconstruction, a fact that may influence the results (24, 74).

Breast symmetry, the timing of the procedure and the individual who evaluates outcomes are also considerations. OS is not always synonymous with the performance of symmetrisation, which improves the cosmetic effects, and time is an important risk factor for the appearance of asymmetries. In patients undergoing BCS, the irradiated breast undergoes little volume change, even with the increase in weight, which is contrary to the contralateral breast, which may present a volume increase and accentuation of ptosis, without alteration of the consistency (24). In patients undergoing mastectomy with prostheses, capsular contracture, the emergence of rippling, elevation of the breast furrow height and, when associated with increased weight, increased asymmetry in the contralateral breast are observed. Such outcomes may influence QOL and breast satisfaction. Regarding the cosmetic result, it is observed that in general, patients are less demanding than health professionals, with disagreement between them regarding the quality of the results, which makes it difficult to compare cosmetic results and QOL (75).

For a long time, the number of QOL questionnaires directed at breast cancer were few and evaluated specific situations, and the breast shape and outcomes were poorly evaluated. For cosmetic breast evaluation we used the BCTOS (43) and MBROS (32). The Breast-Q initially used in plastic surgery has been improved, and new questionnaires specific to the different conditions associated with breast cancer have emerged, allowing better evaluation of the impact of OS in different situations (29, 48, 76). Recently, we started to have the Breast-Q associated with BCS (42), and all questionnaires associated with the Breast-Q are in their second edition (77). The EORTC questionnaires have also evolved in this direction, and the BRECON23 was recently created (46). Updated EORTC QLQ-BR45 (41) aspects associated with breast shape were included, allowing a better evaluation of this aspect as it relates to QOL, but these questionnaires are recent, and the number of publications is limited. The Breast-Q is a questionnaire that has the greatest number of associated publications (49).

Quality of life involves multiple aspects. In breast cancer, there are many sequelae resulted from the treatment (78), many of which are poorly contextualized, since evaluating objective measurements and QOL questionnaires (35). In this context, sequelae associated with shoulder mobility after reconstruction (79), alterations associated with the use of IBBR, or alterations associated with the use of myocutaneous flaps, mainly the rectus abdominis muscle, are observed. Although the degree of satisfaction is high associated with OS, the look under the functional part is little discussed, and me must take care evaluating sequelae and functional functioning. Also, the impact of rehabilitation (80) and exercise (81) in OS is under reported. Future reviews evaluating these conditions are necessary.

The limitation of this study was to group literature results and not evaluate the quality of the studies. Although this situation it was possible to report multiple conclusions. Many studies are needed to accumulate evidence, especially in different populations (39), as the systematic reviews note the presence of heterogeneity in the literature (27, 60–62), a fact possibly associated with the patient selection criteria, techniques used, differences in time since OS, presence of symmetrization and nonuniformity in relation to the questionnaires. Despite these limitations, due to the aforementioned limitations, OS improves QOL. Based on articles presented, we can conclude the following from these studies: (1) Patients undergoing BCS or breast reconstruction have improved QOL compared to those undergoing mastectomy; (2) Patients undergoing BCS or OS have not yet shown improvement in QOL, a fact possibly influenced by patient selection bias; (3) Patients undergoing mastectomy with reconstruction demonstrate better QOL results when reconstruction is performed compared to BCS using autologous flaps and when preserving the nipple-areola complex; (4) Prepectoral implants improves QOL in relation to subpectoral IIBR; (5) ADM do not improves QOL; (6) Patients undergoing prophylactic mastectomy indicate high satisfaction, but patients should be informed about the potential complications associated with the procedure; (7) Satisfaction is high after fat grafting.

Oncoplasty has become a routine procedure. As the literature increases, more publication will occur and new meta-analysis will appear increasing the number of patients for evaluation. The impact of surgery (mastectomy versus mastectomy with fasciocutaneous or myocutaneos flaps for skin closure) in QOL for locally advanced breast cancer was never studied in case-control study, although we believe that this surgery improves QOL (82). Also, new techniques, new indications of EO and robotic surgery are becoming popular, making space for new studies related to QOL. While there is usually a selection bias in studies and the studies are heterogeneous, some results possibly will not change: the preservation of the breast or the nipple-areola complex and the use of autologous flaps are associated with better QOL results. OS already has a defined role in improving QOL.

## Author contributions

RV and AB-J conceived the manuscript. RV, AB-J, IO-J wrote the manuscript. RV, IO-J supervised and edited the manuscript. RV and AB-J share the same authorship. All authors contributed to the article and approved the submitted version.
